# Coupled vs. separate optimization in industry and energy system defossilization analysis: A German case study

**DOI:** 10.1016/j.isci.2025.113381

**Published:** 2025-08-21

**Authors:** Célia Burghardt, Mirko Schäfer, Anke Weidlich

**Affiliations:** 1 INATECH, University of Freiburg, Emmy-Noether-Str. 2, 79110 Freiburg im Breisgau, Germany

**Keywords:** Earth sciences, Environmental science, Energy resources, Energy policy, Energy sustainability, Energy systems, Energy management, Energy Modelling

## Abstract

Achieving carbon neutrality requires defossilizing both the energy system and industry, which are closely linked through shared resources and energy carrier exchange. However, many studies treat them separately, overlooking important feedbacks. This study uses a techno-economic model for German industry to compare coupled and sequential (soft-linked) optimization with the energy system model PyPSA-Eur. Coupled optimization yields similar overall costs (0.3% lower) but different resource use: industry relies more on direct electrification, uses less biomass and hydrogen, and achieves negative emissions (− 24 Mt CO_2_), offsetting energy system emissions. In contrast, the soft-linked approach treats sectoral neutrality independently, requiring more costly direct air capture. This study underscores the role of cross-sectoral feedback in resource and emission allocation and hydrogen use and reveals limitations of sequential approaches in representing these feedbacks.

## Highlights

• Compared coupled and soft-linked optimization of energy and industry sectors
• Coupling reduces direct air capture reliance by enabling negative emissions in industry
• Coupling shifts biomass use from industry to the energy system
• Coupling leads to more direct electrification in industrial transformation

## Graphical abstract

**Figure:**
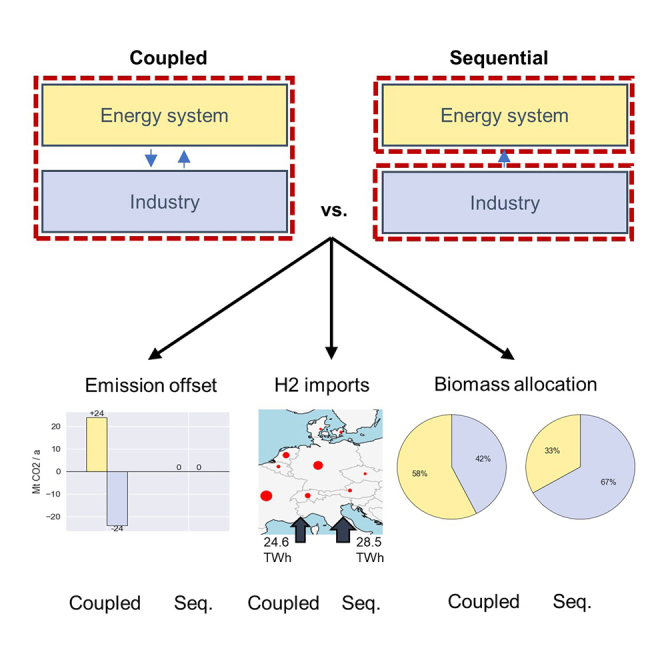

## Introduction

The energy system and industry are major emitters and must defossilize to meet climate goals. They are closely linked: defossilized industry relies on electricity, hydrogen (H_2_), and synthetic fuels to be provided by the energy system, while both compete for limited resources and for remaining emission budgets. Despite this interdependency, optimization studies typically focus on one sector and treat the other sector’s transformation as exogenous by using results from a model of the other sector (sequential approach via soft-link). This method does not fully capture the feedbacks between the sectors. While initial approaches for co-optimization in coupled models (in the context of this study, coupled meaning fully integrated in a joint model, as defined in the study by Helgesen et al.^1^) exist, the impact of coupled versus soft-linked optimization remains unexplored, leaving the influence of integrated transformation unclear.

The following interactions exist between the two sectors. First, the sectors have to jointly meet emission targets and cross-sectoral offsetting of emissions is possible.^2^ Second, industry defossilization options include direct electrification (e.g., electric furnaces) and indirect electrification via H_2_ and synthetic hydrocarbons.^3^ These choices affect capacity and flexibility requirements in the energy system, while energy carrier prices and availability affect cost-minimal industry transformation.^4^^,^^5^ Additionally, limited resources such as biomass and CO_2_ storage are useful in both sectors, and cost-efficient allocation can only be determined when considering trade-offs in both sectors jointly.^3^^,^^5^^,^^6^ The application options for limited resources are summarized in Table 1.

**Table 1:** Application options for shared resources and energy carriers in the model

Resource	Industry	Energy System
Solid biomass (optional BECCS)	Process heat, feedstock after conversion to gas or biofuels	Dispatchable electricity and heat generation, conversion to gas or liquid fuels
Biogas	Process heat, feedstock	Dispatchable electricity and heat generation
CO_2_ storage	Process emissions, emissions from fossil-based process heat	Emissions from fossil gas (space heat, CHP, electricity), temporary storage for synthetic fuels production
H_2_	Process heat, feedstock	Dispatchable electricity and heat generation
Synthetic liquid fuels (methanol, naphtha)	Feedstock for chemicals production	Fuel for transportation and agriculture

See also Table S3.

Sustainable biomass can serve as fuel for process heat and feedstock in industry or for dispatchable power and heat generation in the energy system, and can provide negative emissions when combined with carbon capture (CC) and storage (bioenergy with CC and storage, BECCS).^7^ CO_2_ storage is essential in both sectors, for storing CO_2_ temporarily for synthetic fuel production, or permanently to offset remaining emissions. H_2_ and synthetic fuels are not inherently limited, but their production is energy-intensive, thus their use needs efficient allocation.

These interactions are relevant for policies on carbon management, on the use of limited resources, on sectoral emission budgets and on infrastructure planning. For instance, in Germany, the development of a long-term negative emissions strategy and a carbon management strategy is planned. Cornerstones for these strategies have been formulated,^8^^,^^9^ which set a focus on CC for technologies in the energy system (gas- and biomass-based generators, waste incineration) and industry (hard-to-abate process emissions, e.g., cement and lime),^8^ set the goal to plan a CO_2_ infrastructure,^8^ and identify the need to consider potential conflicts in respect to land use and biomass availability.^9^ Negative emission targets for the years 2030, 2040, and 2045 are planned to be defined based on scenario work.^9^ To define these strategies effectively, it is essential to analyze both conflicts and synergies arising from the parallel transformation of the energy system and industry. Key issues include how to allocate limited resources such as biomass and CO_2_ storage, which shifts in energy carrier demand and supply occur in both sectors, and how remaining emission budgets and CC technologies should be distributed between them.

The transformation of these sectors is studied in normative optimization models that identify cost-minimal climate neutral systems.^10^ In this study, energy system models are defined as models endogenously determining the expansion and operation of conversion, transmission, and storage technologies for energy carriers like electricity, H_2_, and synthetic fuels. Industry models endogenously determine the choice and operation of production routes and process heat technologies.

**Figure 1:**
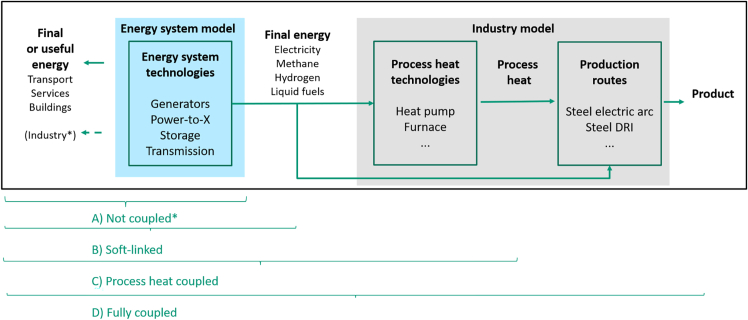
Different degrees of industry sector integration into energy system models (brackets represent parts of the model that are endogenous)

Figure 1 illustrates varying levels of industry integration into energy system models (based on^2^^,^^10^^,^^11^), from treating industry demand as exogenous, taken from literature (A) or a soft-linked industry model (B), to partly (C) or fully coupling (D). Soft-linking approaches are common in large scenario studies for Germany,^12^^,^^13^^,^^14^^,^^15^^,^^16^ and studies at European level.^4^^,^^17^^,^^18^^,^^19^^,^^20^ In these studies, industrial energy and resource demand and emissions are determined first, and given as constraints to the subsequent energy system optimization. Allocation of resources and emissions is done beforehand, based on e.g., today’s sectoral shares,^15^^,^^21^ or giving industry priority^14^^,^^22^ or technical considerations.^20^ The sequential approach does not necessarily reach system-wide cost optima,^2^^,^^23^ but avoids methodological and computational challenges of fully integrating models.^10^

Few studies fully couple industry and energy system models to optimize their transformations simultaneously (D,^2^^,^^5^^,^^23^^,^^24^), and others partially integrate these models by making process heat endogenous but keeping process route choice exogenous (C,^7^^,^^25^^,^^26^). This allows for feedback and optimal resource allocation between sectors, e.g., for biomass.^7^

However, these studies do not compare coupled and soft-linked optimizations, leaving the impact of sectoral interactions versus individual sector optima unclear. A previous comparison focusing only on the subsector pulp and paper suggests that already at this smaller scale sectoral interactions can significantly influence optimal solutions.^2^

This study addresses the gap by comparing cost-optimal transformations of the industry and energy sectors using coupled (D) and soft-linked (B) approaches for Germany. By using identical sectoral models, we isolate the effect of coupling. The aim of this study to understand how the transformation of industry and energy system influences each other when optimized together, answering the following research questions.

(1) How do technology choices and resource and emissions allocations differ between coupled and soft-linked optimization, and which areas are most or least affected?
(2) What limitations of soft-linking hinder accurate representation of cross-sectoral interactions and how can these be better integrated?

**Figure 2:**
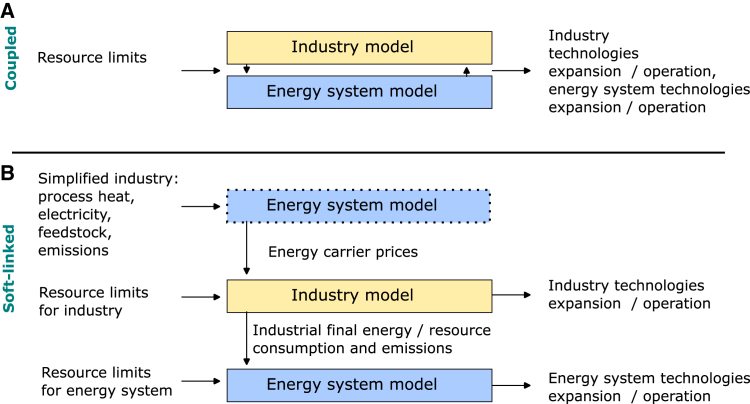
Compared model configurations in this study See also Tables S1 and S2 and Figures S7–S9.

We develop a model of the German energy-intensive industry, comprising options for producing the bulk materials steel, cement, and high-value chemicals (HVC), as well as for providing industrial process heat at different temperature levels. This model is built with the PyPSA framework^27^^,^^28^ and coupled to the energy system model PyPSA-Eur^29^^,^^30^ for Germany and neighboring countries (coupled configuration), or solved independently (soft-linked configuration). The optimization steps of the two configurations are shown in Figure 2: the coupled approach optimizes in a single step, while in the soft-linked approach, energy carrier price time series are obtained from a pre-run of the energy system model, used as input for the industry optimization, and energy system optimization is done in the last step using outputs from the industry optimization. In both configurations, we perform an optimization for the year 2045 under a zero-emission constraint.

The models include the application options for shared resources shown in Table 1. CO_2_ emissions can be captured at point-sources (process routes and process heat in industry; heat, power and combined heat and power (CHP) generators in energy system), obtaining negative emissions when the carrier is biomass, or via direct air capture (DAC). Distributed emissions in the system, such as emissions from product decay or transportation fuels, can be offset by DAC or BECCS. Captured CO_2_ can be either permanently stored or utilized for synthetic fuel production in the energy system.

## Results

### Industry processes and process heat

**Figure 3:**
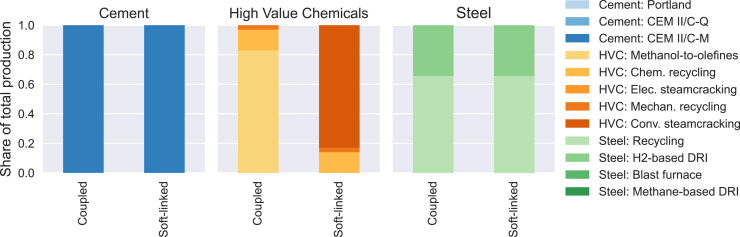
Shares of steel, cement and high value chemicals produced per industrial process route as result of the coupled and soft-linked optimization See also Figure S2 and Tables S5–S7.

The modeled industry sector includes the production of bulk materials and process heat, while product demands are fixed inputs. Figure 3 illustrates the shares of material production through different process routes in the coupled and soft-linked optimizations. Secondary production routes for steel and HVC are limited by secondary material availability (steel scrap and plastic waste) and used to the maximum possible share in both configurations. For plastic recycling, mechanical recycling is prioritized and the sorted out waste fractions are used for chemical recycling. While cement and steel production share identical process routes in both configurations, HVC primary production routes differ. In the coupled optimization, methanol-to-olefines with synthetic methanol is used, whereas conventional steamcrackers with synthetic naphtha and methane are used in the soft-linked optimization. The effects which the industry choices have on the energy system cannot be seen in the sequential approach: In the case of HVC production, the energy system must provide synthetic fuels naphtha or methanol which requires CO_2_ and H_2_ capturing or production, storage and conversion to fuels. Methanol production needs less CO_2_ and hydrogen, but more electricity than naphtha production via Fischer-Tropsch process. In the coupled optimization, these trade-offs can be balanced and the lowest-costs option is methanol-to-olefines from a system perspective.

In the soft-linked optimization, industry is optimized with price time series for different energy carriers as input, such as naphtha, electricity, and hydrogen. These time series are derived from a first energy system optimization (see Figure 2) with an estimated demand for the carriers. Therefore, the price time series approximate prices for the energy system to provide these carriers but are based on a specific demand for these carriers and might change when demand changes, e.g., the price for naphtha might become higher when more naphtha is consumed in the system in total or lower when more electricity is consumed in the system since naphtha production can balance variable renewable generation.

Industrial high-temperature heat is provided by biomass kilns with BECCS in both configurations. Medium-temperature heat supply differs between the configurations: in the coupled configuration, 30 TWh is provided by biomass kilns with BECCS, and 15 TWh by H_2_ kilns. In the separate configuration, all 45 TWh heat come from biomass kilns without BECCS. Low-temperature process heat is provided by electric heat pumps in both configurations. The coupled industry sector combines all biomass kilns for process heat with BECCS, additional to process emissions point-source CC. Thereby, industry reaches negative emissions of −24 Mt CO_2_, which enables some emissions in the energy system and saves DAC expansion. Conversely, the soft-linked configuration offsets industry emissions using BECCS only to the extent that zero emissions are reached. The reason for these differences is that in the soft-linked case, industry is optimized first and does not have an incentive to go below the zero emission limit, since this means additional costs. The cost savings in the energy system in the subsequent step are not known in the industry optimization. Energy- and capital-intensive DAC is left as only option for capturing emissions in the subsequent energy system optimization, since BECCS is ruled out by the fact that all biomass is used up by industry. This is different in the coupled optimization where all technologies to reach zero emissions in both sectors are weighted against each other and combining all biomass usage with CC is identified more cost-efficient than installing DAC.

### Energy system results

The different industry transformations in the configurations set different solution spaces for the energy system optimization. In the soft-linked configuration, the energy system must provide naphtha and methane for steamcracking, for which it chooses Fischer-Tropsch and Sabatier processes requiring H_2_ and CO_2_. This leads to 312 TWh total H_2_ use in Germany for the soft-linked case. In contrast, the coupled configuration uses 296 TWh H_2_ mainly for methanolization, process heat, and H_2_ turbines. The higher hydrogen demand in the soft-linked case is met by higher overseas hydrogen imports across all interconnected countries and by more electrolysis.

For detailed hydrogen and electricity use per technology, see Figure S2 in the supplemental information.

**Figure 4:**
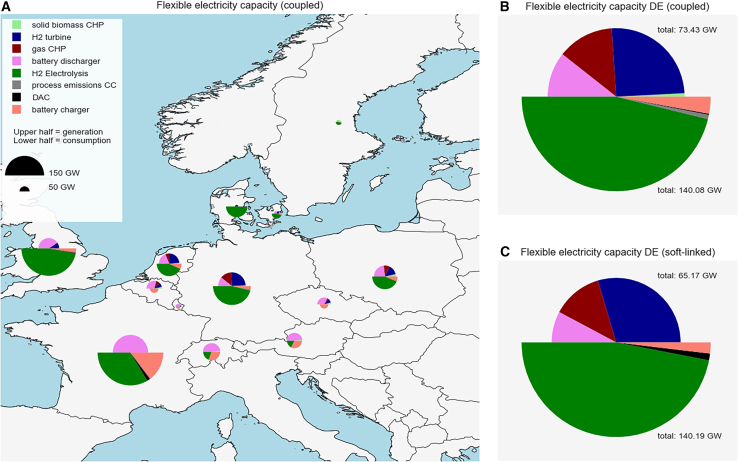
Capacities of flexible electricity generators and consumers in the energy system (A) Capacities for all modeled countries for the coupled configuration. (B) Capacities for the German node DE in the coupled configuration. (C) Capacities for the German node DE in the soft-linked configuration. See also Figures S3–S6.

Despite higher electricity demand from electrolysis in the soft-linked configuration, the coupled case has a greater overall electricity use (1289 TWh vs. 1250 TWh) due to direct electricity demands for processes like methanolization and methanol-to-olefines. Less flexibility is provided by electrolyzers, since less electrolyzer capacities are expanded. Instead, more dispatchable generator capacity is expanded, see Figure 4, mainly in France (123 GW), Germany (73 GW), and Poland (47 GW). The German node expands more flexible generation in the coupled case (73 GW) compared to the soft-linked case (65 GW), while the differences in the other countries are small (see Figure S6 in supplemental information). The flexible generation capacities in the coupled configuration include gas CHPs, batteries, H_2_ turbines, and solid biomass CHPs. Solid biomass CHPs cannot be expanded in the soft-linked case since all solid biomass is consumed by the industry optimization before. Meanwhile, the expansion of fluctuating wind and solar generators shows only minor differences (for details see Figures 3 and 4 in the supplemental information). The higher DAC expansion in the soft-linked configuration leads to a higher demand for low-temperature heat, which leads to higher heat pump expansion and electricity use.

### Limited resources consumption

In both configurations, biomass (solid biomass and biogas) resources are completely consumed, but allocated differently. In the soft-linked case, all solid biomass is used for process heat production, and is unavailable for the energy system in the subsequent step. In the coupled optimization, less solid biomass is used for industry process heat, and 20% is allocated to the energy system in CHPs. Biogas is used by 29% by the industry in the soft-linked optimization (gas for steamcrackers in HVC production, rest in the energy system for boilers and CHPs), and completely for the energy system in the coupled optimization, in which more boilers and CHPs are operated with biogas. Thus, the soft-linked configuration overlooks the system-wide benefit of shifting some biomass usage from industry (and use more expensive alternatives) to the energy system (and prevent more expensive alternatives). The consumption of solid biomass and biogas by technology is shown in Figure S1.

**Figure 5:**
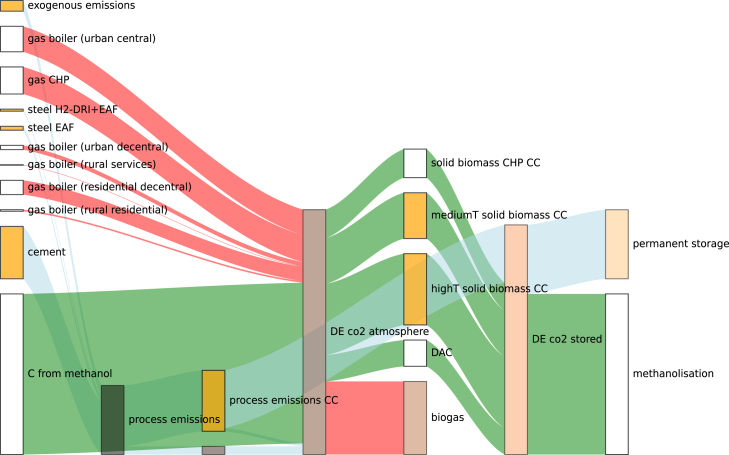
Resulting CO_2_ flows in Germany for the coupled optimization See also Figure S1.

**Figure 6:**
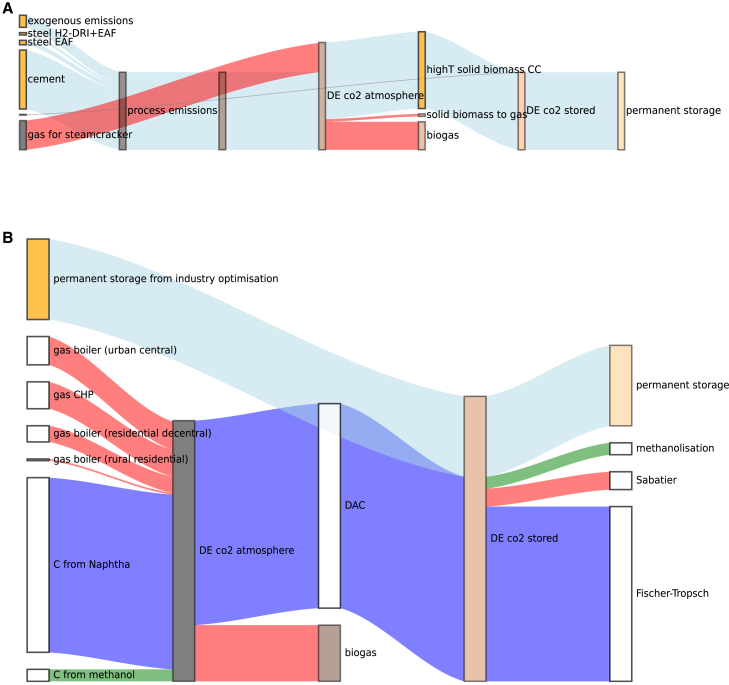
Resulting CO_2_ flows in Germany for the soft-linked optimization (A) CO_2_ flows for the first step industry optimization. (B) CO_2_ flows for the second step energy system optimization. See also Figure S1.

CO_2_ storage capacity is utilized in both configurations for two purposes: permanent storage of process emissions (industry) and temporary storage for synthetic fuel production (energy system). However, the difference between the configurations lies in whether emissions are captured in the industry sector or energy system. Coupled optimization achieves negative industrial emissions (−24 Mt CO_2_), offsetting energy system emissions (Figure 5). Two carbon loops emerge: (1) biogas loop (red flows) capturing CO_2_ from the atmosphere through photosynthesis, which is later released when biogas is burned in boilers or CHPs, (2) methanol loop (green flows) capturing CO_2_ via BECCS in industry process heat technologies, temporarily storing it for later conversion to methanol, and eventually releasing CO_2_ during decay or combustion of methanol-derived products or fuels. The methanol loop is cross-sectoral, since emissions are captured in the industry sector and used in the energy system. In contrast, in the soft-linked configuration, emissions are offset separately within each sector and carbon loops are closed within the sectors (Figure 6). Since solid biomass resources are used up by industry and no more BECCS can be expanded, DAC is left as only option for negative emissions in the energy system for closing the carbon loops (naphtha, methanol, gas), increasing system costs.

### Cost comparison

We compare the yearly costs for 2045 for the result systems of the coupled and soft-linked configurations. The total costs are dominated by capital costs; the only operational costs arise from the consumption of biomass, industry raw materials like limestone and steel scrap, and for ship import of H_2_ and make up 8% (coupled) and 6% (soft-linked) of total costs.

The coupled configuration achieves slightly lower costs (0.3%, or 2.12 b€, see Table 2). The soft-linked configuration reaches lower costs for the industry transformation, since industry is optimized first and costs for the energy system transformation induced by industry transformation are not considered in the industry optimization. The energy system optimization then results in higher yearly costs.

**Table 2:** Comparison of annual costs of the two configurations

	Coupled	Separate	System components
Industry (billion euros)	22.44	22.22	Process routes; process heat; raw material costs; process emissions carbon capture
Energy system (billion euros)	522.12	524.46	Energy carrier conversion, storage, and transmissions; DAC
Total (billion euros)	544.56	546.68	All components

Lower costs in the coupled configuration arise mostly from lower H_2_ imports (−1.0 b€), and lower costs for DAC expansion (−3.3 b€), while the costs for (dispatchable) generator expansion are higher (2.6 b€). Other cost savings come from lower costs for synthetic fuels production and lower costs for low-temperature heat due to lower heat demand from DAC and therefore lower expansion of heat pumps.

### Variations in soft-linked optimization

To find the main drivers of different outcomes in the coupled and soft-linked optimizations, we run input parameter variations in the soft-linked optimization to see if results can approach those of to the coupled optimization results. Two influential factors are examined: the input energy carrier prices and the pre-allocation of resources and CO_2_ emissions to the industry sector.

### Energy carrier price time series

The soft-linked optimization utilizes price time series derived from a pre-optimization step (Figure 2). These prices, representing shadow prices from energy balance constraints for each carrier, node, and time step, aim to reflect system costs for providing energy carriers. However, discrepancies arise because the pre-run assumes an exogenous industrial final energy demand, which differs from the demand resulting from the soft-linked industry optimization. For a comparison of the energy carrier prices and industrial final energy demand, see Figures S7 and S8 in the supplemental information.

To assess the impact of this inconsistency, we conduct a second iteration of the soft-linked configuration, using updated shadow prices from the energy system optimization as input for the industry optimization. The results remained unchanged, indicating that moderate variations in energy carrier prices do not significantly affect industrial optimization outcomes. Even additional price variations for methanol and naphtha (see Figure S9 in supplemental information) had no observable impact, suggesting that price inconsistencies in the soft-linked approach are not a decisive factor.

### Sectoral resource and emission shares

Resource and emissions allocation proved critical in replicating coupled outcomes. By pre-setting biomass (solid biomass and biogas) and CO_2_ emission shares based on coupled results (−24 Mt CO_2_ in industry), soft-linked optimization closely matched coupled outcomes, with minor deviations (e.g., a 9% share of steamcrackers in HVC production). When only biomass shares or only CO_2_ constraints were pre-set, results diverged significantly, so the combination of resource and emission allocation is decisive.

## Discussion

This study demonstrates that coupling industry and energy system optimization leads to defossilization strategies that differ from those derived using separate approaches. While the total costs of the coupled approach are only 0.3% lower (2.11 b€), significant differences arise in biomass allocation, sectoral emissions, and HVC primary production routes. Rather than quantifying the exact cost differences between coupled and soft-linked optimization, the objective of our study was to show the dynamics between the sectors enabled by integrated optimization and highlight areas which are most affected by them. We also wanted to demonstrate how results are affected by methodological choices such as how the models are coupled and how the extra exogenous parameters (endogenous in a fully integrated approach) are chosen.

### Where do interactions between industry and energy system matter?

The interactions between energy system and industry primarily influence technology choices in areas affected by carbon flows, limited resources, and flexible energy demand and supply.

In the coupled configuration, the system-wide optimal allocation of biomass and CC technologies enables negative emissions in the industry sector, which can offset residual emissions in the energy system. In contrast, the sequential approach achieves net-zero emissions separately in each sector, leading to a stronger reliance on DAC in the energy system. This results in differences in the deployment of biomass-based and CC technologies between the two configurations. This leads to differences in technology expansion for biomass-based technologies, and for CC technologies between the two configurations. Additionally, carbon flows are relevant in HVC production, where CO_2_ is required as feedstock, and technology choices differ between coupled and sequential optimization.

Flexible electricity demand and supply differs between coupled and sequential optimization. Industrial hydrogen demand is higher in the sequential optimization, and flexibility can be provided to the energy system with flexible hydrogen production and imports. In the coupled optimization, industry chooses more direct electric routes and flexibility is provided to the energy system by expanding more dispatchable generators.

Areas less affected by sectoral interactions include the expansion of wind and PV generators, as well as steel production, where technology options are not part of carbon flows or resource competition. Low-temperature heat supply for buildings and industry similarly shows minor differences between approaches. Additionally, permanent carbon sequestration is only used for remaining industrial process emissions in both configurations.

### Limitations of soft-linking

Soft-linked optimizations cannot fully replicate the cost-optimal results of coupled approaches. Factors which are endogenous in a coupled approach need to be set exogenously in a soft-linked approach, in the case of our study, energy carrier prices for industry, sectoral shares of resource limits, and sectoral emission shares. These are decisive for the results. While shadow price adjustments for energy carrier prices as one aspect of inconsistency were tested, these did not influence the industry optimization outcomes. The results of variations in soft-linked optimization show that the decisive factor in result differences to the coupled case is the scarce resource allocation which is predetermined in the soft-linked approach and does not match the optimal allocation from the coupled optimization where all options compete. Furthermore, cost-optimal results in the coupled optimization include cross-sectoral emissions offsets and resource competition. These factors which are difficult to account for in soft-linked models. However, soft-linked approaches remain relevant in cases where (1) resources and emissions are allocated beforehand, based on criteria other than system cost optimality or (2) full co-optimization is ruled out by incompatible model structures or excessive run-time. When coupled optimization is not technically possible, the most straightforward approach for finding optimal resource allocation would be to perform several runs of the soft-linked optimization with different allocations of solid biomass, biogas, and emission caps, and compare the total costs for different allocations to find the least-system cost combination. This would require many optimization runs as the search is unstructured. Alternatively, a more directed search can be applied by applying an iterative bi-directional soft-link in which resource limits are expressed via their dual variables, thus, by setting prices instead of hard constraints. This approach was successfully demonstrated by^31^ for generator-capacity expansion and stated to be generally applicable, and thus could be adapted to scarce-resource allocation. In each iteration, the same price for each resource is set in the industry and the energy system optimization and is lowered or increased until the sum of consumption of each resource corresponds to its limit. At this point, the prices have converged to represent the resource limits in the primal problem.

We did not implement the iterative price exchange in this study because our objective was to quantify the gap between one-way soft-linking and full coupling. Future work could assess how far a bi-directional link closes that gap.

### Comparison to previous research

Previous studies on coupled industry and energy system optimization,^2^^,^^5^^,^^23^^,^^24^ underscore the importance of integrating sectoral transformations for optimal resource allocation and emissions abatement. However, these studies do not compare coupled to soft-linked approaches, leaving it unclear whether their results stem from cross-sectoral interactions or represent independent sectoral optima. By explicitly comparing coupled and soft-linked configurations, our study isolates the effects of cross-sectoral feedback.

Focusing on pulp and paper production in Switzerland,^2^ compare coupled versus soft-linked optimization and, as in this study, find contrasting resource allocation and cross-sectoral emission compensation. While the study examines a single subsector with relatively small energy demand and emissions, our study extends the scope by including the most energy-intensive industrial subsectors, making the findings more representative of sectoral interactions.

Cross-sectoral resource allocation has been studied with focus on biomass^7^ and H_2_,^32^ with endogenous use in the energy system and industry process heat technologies. Our study extends their work by making industrial process route choices and thereby industrial energy demand endogenous, which affects biomass and H_2_ use. Furthermore, while^7^ allow for additional, more expensive biomass imports (used in their optimal results), we impose strict biomass availability set to national potentials. They identify carbon provision and negative emissions by BECCS as a main service of biomass usage, which is also a decisive factor in the result differences of the two configurations in this study.

The consistent transformation of the steel and cement sectors and HVC secondary production across configurations highlights areas where model coupling is less critical. Recycling routes and steel production with H_2_-based direct reduction are also observed as preferred process routes in other studies.^12^^,^^13^^,^^15^^,^^16^^,^^23^^,^^24^ In primary HVC production, the choice of process routes is less consistent across the cited studies and shows a mix of methanol-to-olefines and steamcracking, with diverse origins of the required liquid fuels.

### Implications for modelers and policymakers

Our study showed areas in which interactions between energy system and industry are most relevant. These are sectoral choices affected by biomass allocation, carbon loops, and sectoral shares in residual and negative emissions, and flexibilities in the energy system by synthetic fuel production or dispatchable generators. Modelers focusing on these areas should consider sectoral interactions and be aware of the differences between soft-linking and coupling models. According to our study, one-directional soft-linking would be an inadequate approach for these cases, and in cases where full integration is not possible, an iterative bi-directional soft-linking approach may be more suitable. On the other side, this study also showed areas less affected by cross-sectoral interactions, for which sequential approaches are sufficient.

This paper also highlights that assumptions or inputs have to be made in soft-linked models, and that these are decisive for the outcomes. They should therefore be clearly stated as inputs and variations of them be tested.

Policies touching upon the areas identified relevant for sectoral interactions should be designed with an integrated view of energy system and industry, weighting options in both sectors against one another. Examples for such policies are in the field of carbon management, strategies for biomass use, planning of flexibilities in the energy system, and infrastructure planning for hydrogen and CO_2_ networks connecting sources and uses in both sectors. National strategies for carbon management and negative emission technologies are currently under development for Germany.^8^^,^^9^

### Limitations of the study

The spatial resolution of one node per country overlooks variations in renewable energy potentials and transport constraints for energy carriers within countries. While networks for cross-border exchange of hydrogen and electricity are endogenous to the model, no infrastructure constraints are considered within the countries. Increasing spatial resolution would bring additional insights to the research questions by considering heterogeneous renewable potentials, locations of electricity technologies, and industry processes and transportation constraints. Furthermore, the national scope of the study could be extended to European scope, considering heterogeneous industrial structures and conditions for renewables.

The use of an overnight, greenfield optimization does not consider the transformation from the current system toward climate-neutrality and does not represent effects of currently installed technologies, timing of transformation and limits of expansion speed for energy system^17^ and industry.^33^ Future work could study feedbacks between the two sectors along their defossilization pathways.

This study assumes flat profiles for industrial processes, corresponding to continuous operation at full capacity. No load shifting or demand response of industrial processes is included, making electric processes inflexible which means flexibilities must be built elsewhere in the system (e.g., dispatchable generators). Industry processes based on indirect electrification (through synthetic energy carriers) run continuously as well, but indirectly provide flexibility because the production of the fuels is flexible, especially electrolysis. Incorporating the option for industry to shift loads in the model would add a further interaction between energy system and industry where synergies can be found and may make direct electric processes more favorable for the system.

For industrial processes, continuous operation at full capacity is assumed and no load shifting option is modeled. Hence, direct-electric routes (e.g., electric arc furnaces) cannot shift load to periods of high renewable availability, and the system must procure flexibility elsewhere (e.g., batteries, H_2_ turbines). Industry processes based on indirect electrification (through synthetic energy carriers) run continuously as well, but indirectly provide flexibility to the system because the production of the fuels can be operated flexibly. Allowing load shifting for industry processes could allow further synergies with the energy system and change the attractiveness of direct electric routes.

Finally, resource limits of the energy and industry sectors beyond biomass use, CO_2_ storage and CO_2_ emissions are not considered in this study. Land use is indirectly considered by limiting expansion of wind and solar generators at every node according to regional land use restrictions. Future work could include further environmental impact categories, e.g., water use, where energy system technologies and industry processes have strong impacts^34^ and compete for limited resources.

## Resource availability

### Lead contact

Further information and resources can be provided upon request to Célia Burghardt (celia.burghardt@inatech.uni-freiburg.de).

### Materials availability

The study did not generate new unique reagents.

### Data and code availability

• All data used to run the model and replicate the study results are from publicly available sources. These data have been compiled and deposited in a general-purpose repository and are publicly available as of the date of publication at Zenodo: https://doi.org/10.5281/zenodo.14645227.
• All original code has been deposited at both Zenodo and GitHub and is publicly available as of the date of publication. Zenodo: https://doi.org/10.5281/zenodo.14645227; GitHub: https://github.com/celia-burghardt/Industry_optimisation_model/tree/main.
• Any additional information required to reanalyze the data reported in this paper is available from the lead contact upon request.

## Acknowledgments

This work is funded by a doctoral grant by the 10.13039/100007636German Federal Environmental Foundation (10.13039/100007636DBU).

## Author contributions

Conceptualization, C.B., M.S., and A.W.; methodology, C.B. and M.S.; software, C.B.; data curation, C.B.; formal analysis, C.B.; visualization, C.B.; writing – original draft, C.B.; writing – review and editing, C.B., M.S., and A.W.; funding acquisition, C.B. and A.W.; supervision, M.S. and A.W.

## Declaration of interests

Authors declare no competing interests.

## Declaration of generative AI and AI-assisted technologies in the writing process

During the preparation of this work, the authors used ChatGPT for improving language. They reviewed and edited the content afterward and take full responsibility for the content.

## STAR★Methods

### Key resources table

**Table:** 

REAGENT or RESOURCE	SOURCE	IDENTIFIER
**Software and algorithms**		

Industry model	This paper and corresponding code on Zenodo and Github	https://doi.org/10.5281/zenodo.14645227 https://github.com/celia-burghardt/Industry_optimisation_ model/tree/main
PyPSA framework	Brown et al.^27^ (Journal paper) Brown et al.^28^ (Documentation)	https://doi.org/10.5334/jors.188 https://pypsa.readthedocs.io/en/latest/index.html

**Other**		

PyPSA-Eur energy system model	Hörsch et al.^29^ (Journal paper) Brown et al.^30^ (Documentation)	https://doi.org/10.1016/j.esr.2018.08.012 https://pypsa-eur.readthedocs.io/en/latest/
JRC-ENSPRESO database	Ruiz et al.^35^	https://data.jrc.ec.europa.eu/collection/id-00138
IDEES database	Joint Research Center,^36^	https://data.jrc.ec.europa.eu/collection/id-0110

### Method details

We build two configurations of the same sectoral models: a coupled and a soft-linked configuration (see Figure 2).

The industry model characterizes each process route and process heat technology option with specific emissions, energy carrier and process heat use, and raw materials (e.g., steel scrap or plastic waste for the recycling routes), and operating and annualized investment costs. Model inputs are bulk material demands (steel, cement, HVC) and aggregated demands for less energy-intensive sectors based on 2021 data from the IDEES database^36^ and from.^37^ The demand for process heat is partly endogenous because it depends on the operated process routes.

The energy system model represents Germany and countries with a direct power transfer link, which is the same scope as used in the PyPSA-Ariadne version focusing on Germany,^38^ at a resolution of one node per country. Technologies for the conversion, transmission, and storage of multiple energy carriers (electricity, H_2_, methane, naphtha and oil, methanol) and heat are expanded and operated. Hydrogen can be generated by electrolysis and exchanged between the countries via pipelines, or imported via ship. No supply restrictions for hydrogen imports are set. Also, supply chain risks for technology availability are not considered. In the coupled case, the exogenous industrial energy demand at the German node is replaced by attaching the industry model, while in the soft-linked case, it is replaced by energy demand resulting from industry optimization. The energy carrier and process heat demands for industry sectors other than steel, cement and HVC production are exogenous inputs in both configurations. The energy carrier demand for buildings (electricity and low-temperature heat demand) and transportation (electricity and liquid fuels) are exogenous in the model, while technologies to provide space heat, electricity and liquid fuels, considered part of the energy system in this study, are expanded and operated endogenously. These technologies thus compete for biomass, CO_2_ storage, CO_2_ budget and hydrogen and synthetic fuels, along with the industry sector and other energy system technologies, and their expansion is system cost optimal. For the transportation sector, road transport is assumed to be fully electric in 2045 and a fixed liquid fuel demand for aviation, shipping and agricultural machines is set, which can be provided by synthetic fuel production in the system or by fossil fuels.

Limits for solid biomass and biogas are taken from the JRC-ENSPRESO database, scenario ENS-Med for the year 2040.^35^ The potentials are limited to residual biomass, and exclude bioenergy crops and biomass imports. The CO_2_ emission limit is set to zero, and Germany must also reach zero emissions individually.

The following sections describe the optimization problems of the coupled and soft-linked configurations. A full model formulation and a list of variables (Table S1), parameters (Table S2) and sets (Table S3) is given in the supplemental information. The optimizations minimize annual system costs for expansion and operation of technologies, under emission and resource caps, in an overnight (no pathway), greenfield (no current technology capacities) approach for the year 2045.

#### Coupled configuration

In the coupled configuration, industry and energy system are integrated through energy balances and shared emissions and resource constraints.

The objective function (1) minimizes total system costs over all time steps t∈T and nodes n∈N, consisting of costs of the industry (3) and the energy system (2). Decision variables are the expansion ${\bar{i}}_{p,n}$ and operation $i_{p,n,t}$ of industry processes p∈P, expansion $\bar{g}$ and operation $g_{k,n,t}$ of energy system technologies k∈K (conversion and storage technologies), and expansion of energy transmission capacity ${\bar{l}}_{e,n,m}$. The total costs consist of annualized investment costs, operation costs and costs for consuming resources *r*, with specific investment $C_{a}^{\text{inv}}$, operational $C_{a}^{\text{op}}$, and resource costs (consumption $R_{a}^{\text{ind}}$ times price $C_{r}^{\text{res}}$) defined for each technology a∈A,A=P∪K. The operational costs do not include energy costs, since energy demands induce costs indirectly for their supply.

(Equation 1) $$\min_{g,i,\bar{g},\bar{l},\bar{i}}\;z^{esm}+z^{ind}$$

with:

(Equation 2) $$z^{esm}=\sum_{k\in K}\;\sum_{n\in N}\left(\sum_{t\in T}g_{k,n,t}\left(C_{k}^{\text{op}}+\sum_{r\in R}R_{k,r}C_{r}^{\text{res}}\right)+{\bar{g}}_{k,n}C_{k}^{\text{inv}}\right)+\sum_{e\in E}\sum_{n,m\in N}{\bar{l}}_{e,n,m}C_{e,n,m}^{\text{inv},\text{tr}}$$

(Equation 3) $$z^{ind}=\sum_{p\in P}\;\sum_{n\in N}\;\sum_{t\in T}i_{p,n,t}\left(C_{p}^{\text{op}}+\sum_{r\in R}R_{p,r}C_{r}^{\text{res}}\right)+\sum_{n\in N}\;\sum_{p\in P}{\bar{i}}_{p,n}C_{p}^{\text{inv}}$$

(Equation 4) $$D_{e,n,t}=\sum_{p\in P}i_{p,n,t}\cdot E_{p,e}+\sum_{k\in K}g_{k,n,t}\cdot E_{k,e}+\sum_{m\in N}\left(l_{e,m,n,t}-l_{e,n,m,t}\right),\forall e\in E,\forall n\in N,\forall t\in T$$

(Equation 5) $$D_{m,n,t}^{\text{mat}}=\sum_{p\in P}i_{p,n,t}\;\cdot \;M_{p,m}\;\forall t\in T,\forall n\in N,\forall m\in M$$

#### Soft-linked configuration

This approach sequentially optimizes industry and energy system. Industrial final energy demand as output from the industry optimization is fed to the energy system optimization. This configuration requires additional inputs compared to the coupled configuration.

Unlike the coupled optimization, where industrial energy costs emerge indirectly through the required energy system transformation, soft-linked optimizations require price time series $\lambda_{e,t}$ for all energy carriers. These should ideally represent the costs for the energy system to provide the energy carriers. Therefore, prices are derived from a pre-run of the energy system model, interpreting the shadow prices (dual variables) of energy balance equations for each node and energy carrier as price time series. In this pre-run energy system model, industry is represented in a simplified manner: industrial energy demand (process heat, electricity, and feedstock) reflects today’s energy balances^36^ with some adjustments aligned with the current industry representation in the PyPSA-Eur model.^29^^,^^30^

In soft-linked optimizations, resource limits must be pre-split between sectors as allocation cannot occur endogenously. To maximise the industry sector’s solution space, we do not impose additional resource constraints, effectively granting it demand-sector priority access. This restricts the energy system to use only leftover resources not consumed by the previous industry transformation (see Table 1).

(Equation 6) $$\min_{i,\bar{i}}\;z^{\text{ind}}+\sum_{p\in P}\;\sum_{e\in E}\;\sum_{n\in N}\;\sum_{t\in T}i_{p,n,t}\;\cdot \;E_{p,e}\;\cdot \;\lambda_{e,t}$$

(Equation 7) $$\min_{g,\bar{g}}\;z^{\text{esm}}$$

#### Modeled carbon flows

CO_2_ is emitted at point-sources (production processes and process heat in industry, and combustion-based electricity and heat generators in the energy system) and distributed (from product decay or combustion of transportation fuels). Point-source emissions from can be captured at the source, which is less energy-intensive than DAC. Capture options from the atmosphere, available in both sectors, are DAC and BECCS. BECCS can be applied in the industry (biomass process heat) and the energy system (biomass CHPs or electricity generation), as in.^7^

Captured CO_2_ can be either permanently stored or utilized for synthetic fuel production. In the latter case, the carbon is released back into the atmosphere upon fuel combustion or product decay, completing a carbon loop.
