# Supplementary material for: Coupled vs. separate optimization in industry and energy system defossilization analysis: A German case study
Source: iScience. 2025 Aug 21;28(9):113381. doi: 10.1016/j.isci.2025.113381 (PMC12450739; doi:10.1016/j.isci.2025.113381)
Supplement: Document S1. Figures S1–S9, Tables S1–S8, and Methods S1 [file mmc1.pdf]

iScience, Volume *28*

Supplemental information

Coupled vs. separate optimization in industry and energy system defossilization analysis: A German case study

Célia Burghardt, Mirko Schäfer, and Anke Weidlich

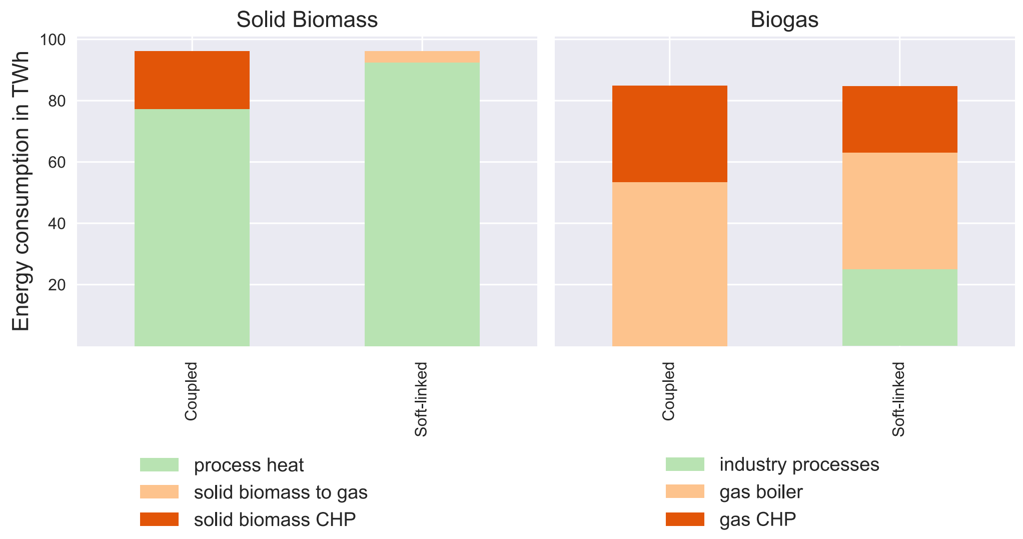

Figure S1: Resulting biogas and solid biomass consumption at the German node in the energy system (orange shades) and in the industry sector (green shades). Left: coupled configuration. Right: soft-linked configuration. Related to fig. 5-6.

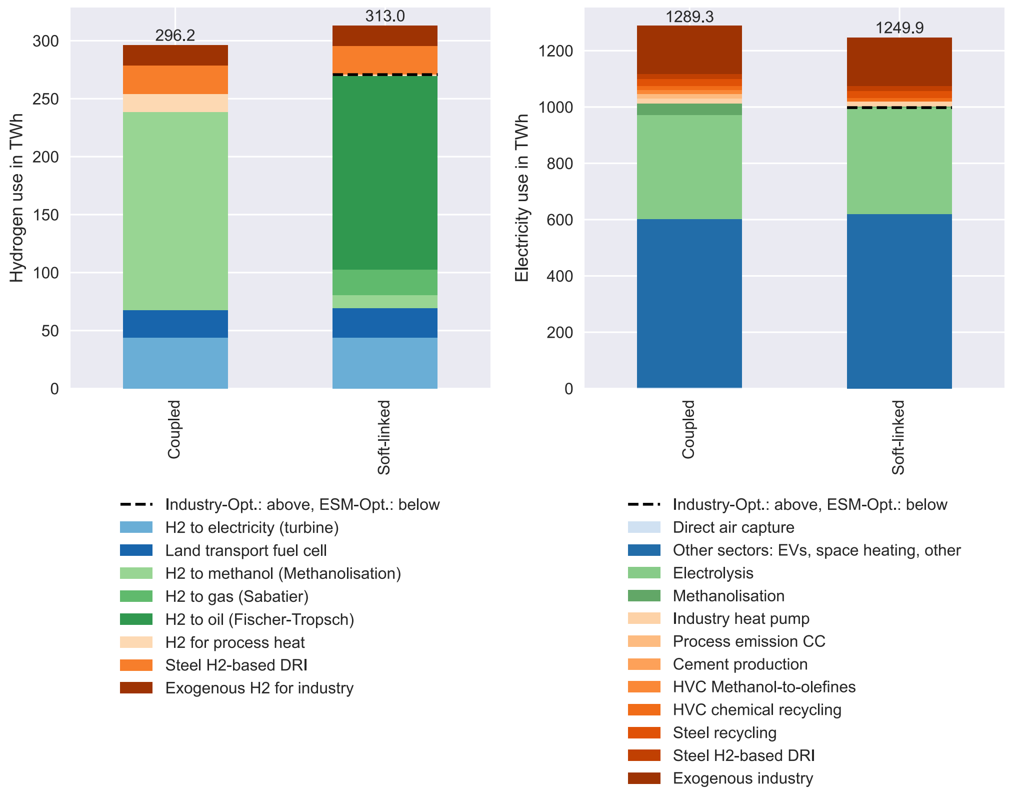

Figure S2: Hydrogen and electricity consumption by technology at the German node in the coupled and soft-linked configurations. Left: Hydrogen consumption. Right: electricity consumption. Related to fig. 3.

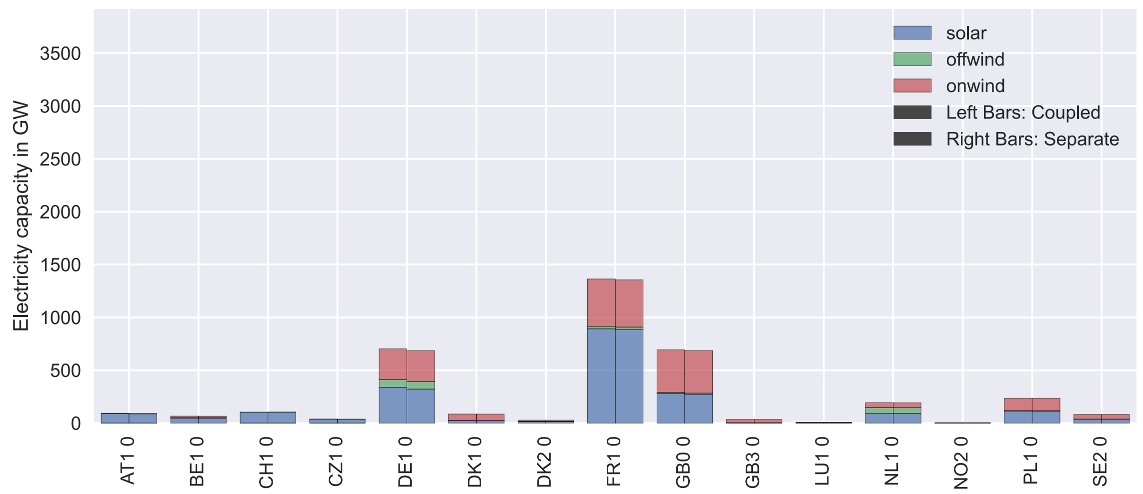

Figure S3: Renewable capacities in coupled and soft-linked configurations per node. Related to fig. 4.

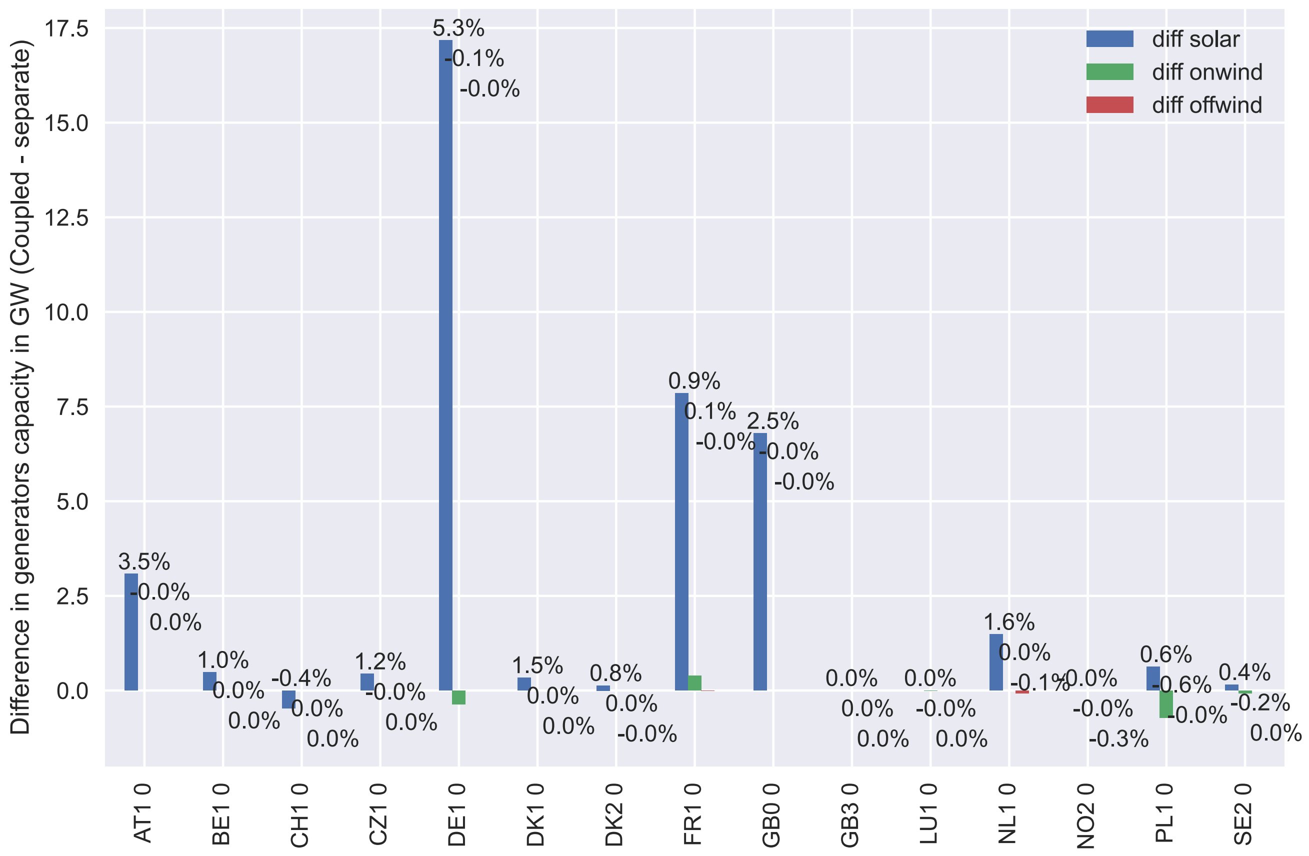

Figure S4: Difference in renewable capacities in coupled and separate configurations per node. Related to fig. 4.

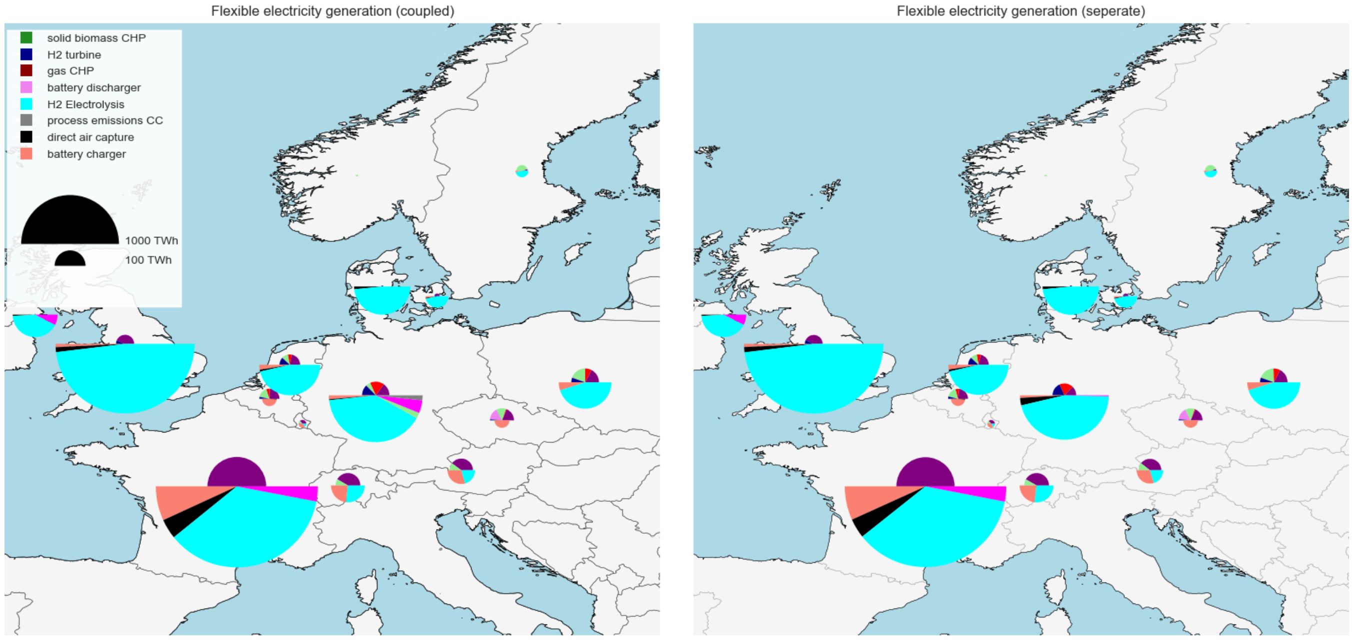

Figure S5: Electricity generation and consumption of flexible generators (upper half of circles) and consumers (lower half of circles). Left: coupled configuration. Right: soft-linked configuration. Related to fig. 4.

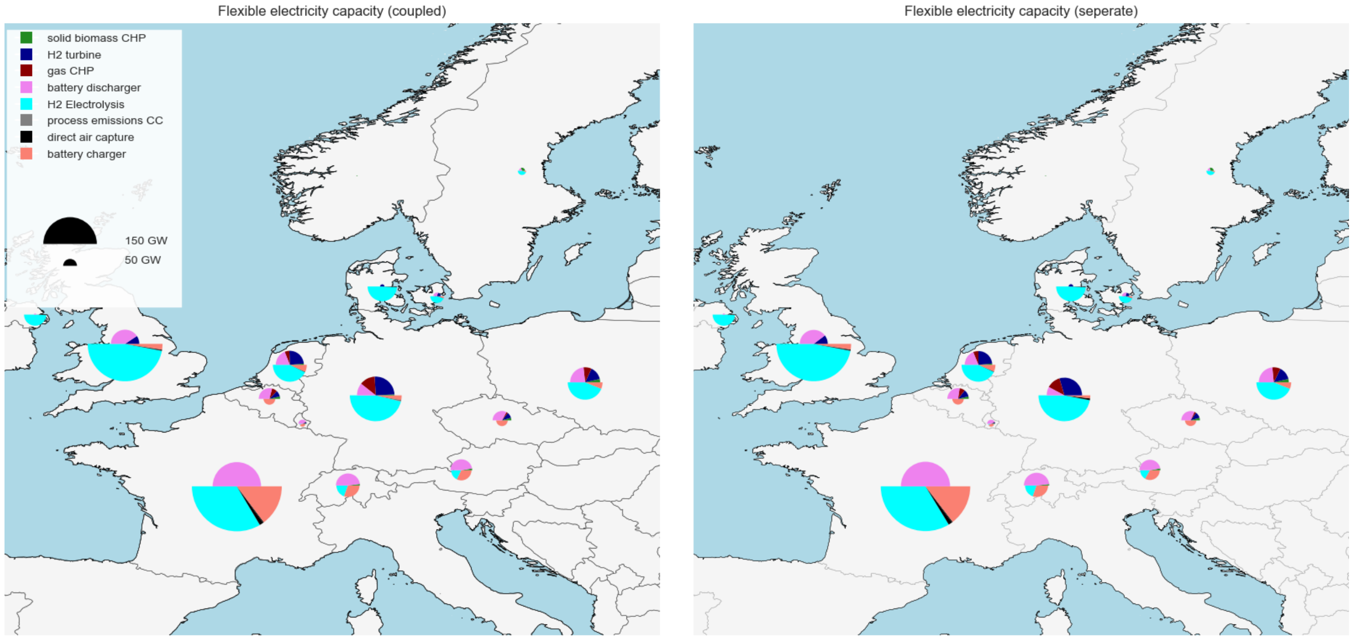

Figure S6: Flexible capacity for electricity generation (upper half of circles) and consumption (lower half of circles). Left: coupled configuration. Right: soft-linked configuration. Related to fig. 4.

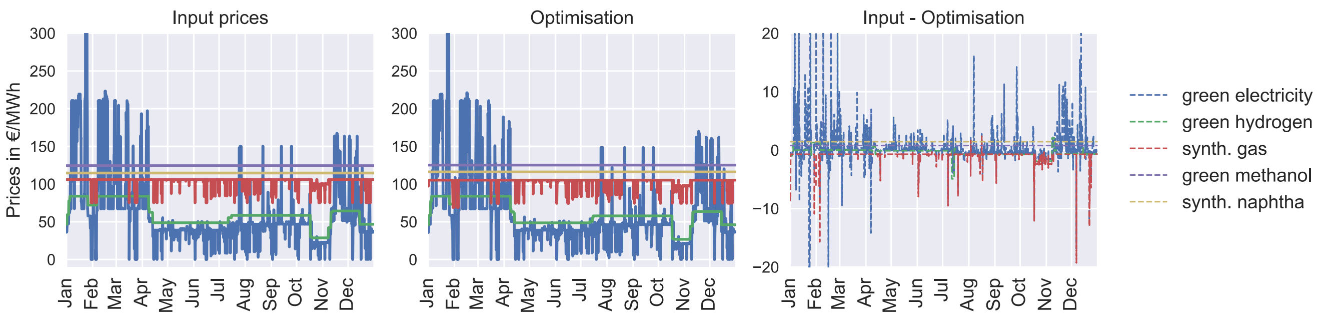

Figure S7: Comparison of input energy carrier prices and energy carrier shadow prices in the soft-linked configuration at the German node. Left: Input prices in the industry optimization derived from shadow prices in the pre-optimization. Middle: Energy carrier shadow prices of the energy balances resulting from the energy system optimization in the second step. Right: Input energy carrier prices of the industry optimization minus shadow prices of the energy system optimization. Related to STAR methods and Methods S1.

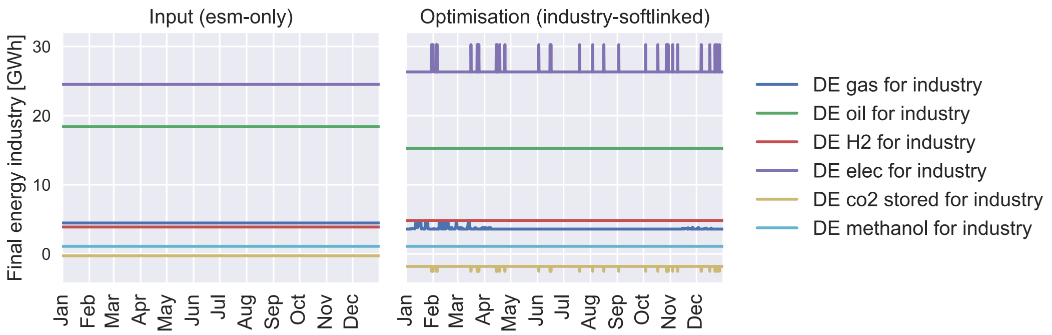

Figure S8: Comparison of input industrial final energy demand of the pre-optimization and output industrial final energy demand of the industry optimization. Left: Input industrial final energy demand. Right: Resulting industrial final energy demand from the industry optimization. Related to fig. 2.

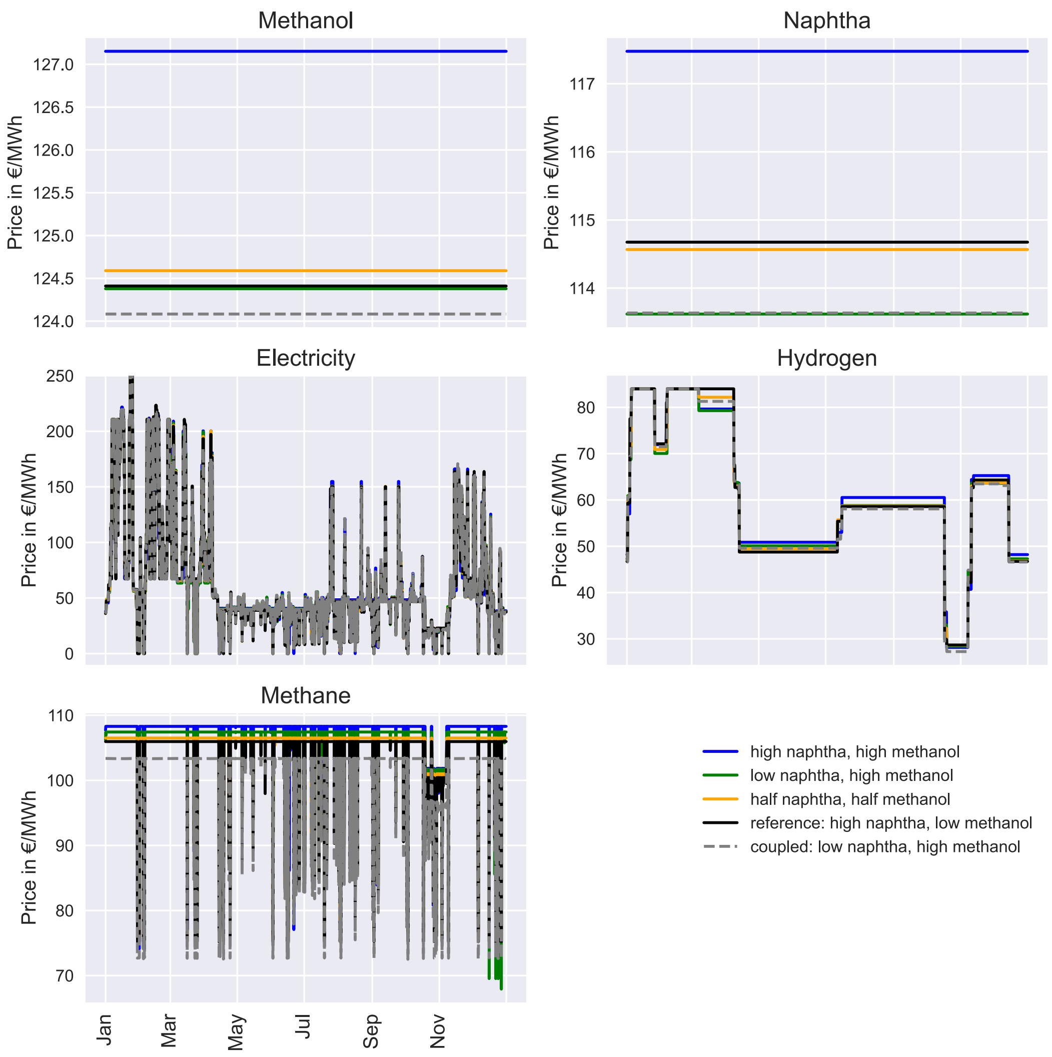

Figure S9: Input energy carrier prices for the reference and variation cases of the soft-linked configuration, derived from shadow prices in the pre-optimization under high, half, and low methanol and naphtha demand, corresponding to the demand for supplying all, half, or none of primary high-value chemicals via processes using these carriers. A) Methanol price time series. B) Naphtha price time series. C) Electricity price time series. D) Hydrogen price time series. E) Methane price time series. Related to fig. 2 and STAR methods.

Table S1: Model variables. Related to STAR methods and methods S1.

**Variable**	**Index Sets**	**Description**	**Unit**
$\overline{g}$	$\mathcal{K}, \mathcal{N}$	Installed capacity of energy system technologies	MW
$\overline{l}$	$\mathcal{E}, \mathcal{N}, \mathcal{N}$	Transmission line capacity node to node	MW
$\overline{i}$	$\mathcal{P}, \mathcal{N}$	Installed capacity of industrial processes	MW
g	$\mathcal{K}, \mathcal{E}, \mathcal{N}, \mathcal{T}$	Operation of energy system technologies	MWh
i	$\mathcal{P}, \mathcal{N}, \mathcal{T}$	Industry processes operation	t
l	$\mathcal{E}, \mathcal{N}, \mathcal{N}, \mathcal{T}$	Transmission of energy carrier from first node to second node	MWh
z^{ind}	-	Annual costs of industry	€
z^{esm}	-	Annual costs of energy system	€

Table S2: Model parameter for all optimizations, for the industry-only optimization and for the energy system(ES)-only optimization. Related to STAR methods and methods S1.

Parameter	Index Sets	Description	Unit
C^{op}	$\mathcal{A}$	Operating cost of technologies	€/MWh or €/t
C^{inv}	$\mathcal{A}$	Investment cost of technologies	€/MW or €/t
C^{res}	$\mathcal{R}$	Price of resources	€/MWh or €/t
$C^{inv,tr}$	$\mathcal{E},\mathcal{N},\mathcal{N}$	Investment cost for transmission	€/MW
R	$\mathcal{A},\mathcal{R}$	Resource demand for technology operation	MWh/MWh or t/MWh
R_r^{max}	$\mathcal{R}$	DE resource limit	t or MWh
$R_r^{max,tot}$	$\mathcal{R}$	Overall resource limit	t or MWh
E	$\mathcal{A},\mathcal{E}$	Energy demand of technology operation	MWh/MWh or MWh/t
M	$\mathcal{P},\mathcal{M}$	Material production of industry process	t/MWh
D	$\mathcal{E},\mathcal{N},\mathcal{T}$	Energy demand	MWh
D^{exo}	$\mathcal{E},\mathcal{N},\mathcal{T}$	Exogenous energy demand (non-bulk industry)	MWh
D^{mat}	$\mathcal{M},\mathcal{N},\mathcal{T}$	Exogenous material demand	t
X	$\mathcal{A}$	Emissions of technology operation	t/MWh and t/t
$\overline{G}^{max}$	$\mathcal{K},\mathcal{N}$	Capacity limit of generator k at n	MW
$\overline{L}^{max}$	$\mathcal{E},\mathcal{N},\mathcal{N}$	Capacity limit of transmission from m to n	MW
CF	$\mathcal{K},\mathcal{N},\mathcal{T}$	Generator capacity factor	-
λ	$\mathcal{E},\mathcal{T}$	Industry-only: Price of energy carriers	€/MWh
D^{ind}	$\mathcal{E},\mathcal{N},\mathcal{T}$	ES-only: Industrial final energy demand	MWh/t
R^{ind}	$\mathcal{R}$	ES-only: Resource demand for industry	t/t

Table S3: Model sets and their elements. Related to STAR methods and method S1.

Set	Description	Elements
$\mathcal{P}$	Industrial processes	’steel EAF’, ’steel H2-DRI+EAF’, ’steel ISW’, ’steel NG-DRI+EAF’, ’hvc steamcracker’, ’hvc chemical recycling’, ’hvc mechanical recycling’, ’hvc electric steamcracker’, ’hvc MtO’, ’cement CEM I’, ’cement CEM II/C-M’, ’cement CEM II/AB-M’, ’cement CEM II C/Q-L’, ’lowT industry solid biomass’, ’lowT industry solid biomass CC’, ’lowT industry methane’, ’lowT industry methane CC’, ’lowT industry heat pump’, ’lowT industry electricity’, ’solid biomass for mediumT industry’, ’solid biomass for mediumT industry CC’, ’gas for mediumT industry’, ’gas for mediumT industry CC’, ’hydrogen for mediumT industry’, ’gas for highT industry’, ’gas for highT industry CC’, ’hydrogen for highT industry’, ’process emissions CC’, ’gas for industry CC’, ’mediumT industry electricity’, ’plasma for highT industry’, ’solid biomass for highT industry’, ’highT industry solid biomass CC’, ’coal for highT industry’, ’waste for highT industry’
$\mathcal{M}$	Materials	Steel, cement, HVC
$\mathcal{T}$	Timesteps	0, 3, ..., 2920 (1 year in 3h resolution)
$\mathcal{N}$	Nodes in the system	1 node per country for Germany + countries with direct power links
$\mathcal{K}$	Energy technologies	’OCGT’, ’H2 Electrolysis’, ’H2 Fuel Cell’, ’Sabatier’, ’SMR CC’, ’SMR’, heat pump, resistive heater, gas boiler, gas CHP’, gas CHP CC’, ’biogas to gas’, ’solid biomass CHP’, ’ solid biomass CHP CC’, ’biomass boiler’, ’biomass to liquid’, ’BioSNG’, ’methanolization’, ’Fischer-Tropsch’, ’DAC’, ’BioSNG CC’, ’solid biomass to hydrogen CC’, ’biomass to liquid CC’, ’electrobiofuels’, ’solid biomass to electricity’, ’solid biomass to electricity CC’, ’waste CHP CC’, ’waste CHP’, ’H2 turbine’; onshore wind, offshore wind, solar, solar rooftop, run-of-river, hydropower; charge and discharge of stores for: ’co2 store’, ’gas store’, ’H2 store’, ’battery’, ’water tanks heat store’, ’methanol store’, ’oil store’, ’home battery’, ’highT industry heat store’, ’mediumT industry heat store’, ’lowT industry heat store’
$\mathcal{R}$	Resources	’co2 stored’, ’biogas’, ’solid biomass’, ’municipal solid waste’, ’plastic waste’, ’light packaging waste’, ’steel scrap’
$\mathcal{E}$	Energy carriers and heat	electricity, methane, hydrogen, space heat (residential / servives; central / decentral), industry process heat (’lowT industry’, ’mediumT industry’, ’highT industry’), methanol, oil / naphtha, coal
$\mathcal{E}^{tr}$	Transmittable en. carriers	Electricity, hydrogen
$\mathcal{A}$	All technologies	$\mathcal{K} \cup \mathcal{P}$

Table S4: Steel production process parameters taken from[1] (material demand, captial costs) and[2] (energy carrier demand) and operational costs calculated based on raw material demand and prices. Related to STAR methods and methods S1.

	**Electric arc**	**H2 - direct iron reduction + electric arc**	**Blast-Basic oxygen furnace**	**methane-direct iron reduction + electric arc**
Electricity in MWh/t	0.905	1.227	0.886	1.227
Biomass in MWh/t	0.000	0.000	0.000	0.000
Methane in MWh/t	0.000	0.000	0.297	1.700
Hydrogen in MWh/t	0.000	1.700	0.000	0.000
Process heat highT in MWh/t	0.08	0.08	0.00	0.08
Coal in t/t	4.933	4.933	4.933	4.933
Iron ore in t/t	0.000	0.000	1.390	0.000
Iron ore pellets in t/t	0.000	1.460	0.000	1.460
Steel scrap in t/t	1.026	0.170	0.170	0.170
Graphite electrode in t/t	0.003	0.002	0.000	0.002
Lime in t/t	0.280	0.050	0.278	0.050
Process emission in t/t	0.03	0.03	0.68	0.03
Capital cost in €/(t/y)	17.00	39.00	16.00	39.00
Operational cost in €/t	328.65	302.89	394.61	302.89
Production 2021 in kt	12941.41	0.00	27124.88	0.00

Table S5: Parameter for HVC process routes. Related to STAR methods and methods S1.

	**Steam-cracker**	**Chemical recycling**	**Mechanical recycling**	**Electric steam-cracker**	**Methanol-to-olefines**
Electricity	0.16	6.90	0.55	4.86	1.39
Coal	0.04	0.00	0.00	0.04	0.00
Methane	2.89	0.00	0.00	0.00	0.00
Naphtha	12.31	0.00	0.00	12.31	0.00
Methanol	0.00	0.00	0.00	0.00	12.96
Plastic waste	0.00	2.39	3.65 (1.39 residual)	0.00	12.96
Capital cost per t/y	27.00	141.00	82.00	27.00	28.00
Production 2020 in kt	13089.00	0.00	0.00	0.00	0.00
**Sources**	[2,3]	[4,5]	[4,6]	[2,3,7]	[7,8]

Table S6: Cement process routes parameter taken from[2,9,10], capital costs from[11] and operational costs calculated from raw material demand and prices and process heat demand and emissions calculated from clinker content. Related to STAR methods and methods S1.

	CEM I	CEM II/C-M	CEM II/AB-M	CEM III/A	CEM II C/Q-L
Electricity in MWh/t	0.08	0.04	0.06	0.04	0.04
Process emissions in t/t	0.70	0.37	0.55	0.37	0.37
Process heat	0.04	0.02	0.03	0.02	0.02
Process heat highT in MWh/t	0.56	0.30	0.44	0.30	0.30
Clinker in t/t	0.95	0.50	0.74	0.50	0.50
Limestone excl. limestone for clinker in t/t	0.00	0.20	0.00	0.00	0.15
Supplementary cementitious materials, excl. blast furnace slag and limestone in t/t	0.00	0.25	0.20	0.00	0.00
Blast furnace slag in t/t	0.00	0.00	0.00	0.45	0.00
Other in t/t	0.05	0.05	0.05	0.05	0.05
Calcinated clays in t/t	0.00	0.00	0.00	0.00	0.30
Limestone in t/t	0.95	0.70	0.74	0.50	0.65
Operational cost in €/t*	12.37	17.95	16.94	28.69	65.56
Capital costs in €/(t/y)	14.00	8.00	11.00	8.00	8.00

Table S7: Share of HVC and steel production going to products in enduse sectors, average lifetime of the products, and waste recovery rate per enduse sector, based on[12],[13],[14],[15],[16]. Related to STAR methods and methods S1

**Enduse**	**Share of produced steel**	**Share of produced HVC**	**Average lifetime (years)**	**Recovered share**
Transportation	0.3	0.11	13	0.82
Mechanical engineering	0.1	0	20	0.87
Construction	0.47	0.25	50	0.74
Other products	0.13	0.27	10	0.58
Packaging	0	0.31	1	0.7
Electronics	0	0.06	5	0.2

Table S8: Raw material prices for the year 2020. Related to STAR methods and methods S1.

**Material**	**Price in €/t**	Source
Coal	110.00	[1]
Coke	200.00	[1]
Waste polymer	0.00	[17]
Blast furnace slag	49.29	[10]
Lime	100.00	[1]
Calcinated clays	190.33	[10]
Recycled concrete	32.55	[10]
Clay	15.81	[10]
Gypsum crude	11.16	[10]
Limestone	13.02	[10]
Supplementary cementitious materials (average price), excl. blast furnace slag and limestone	35.34	[10]
Iron ore	114.00	[1]
Iron ore pellets	154.00	[1]
Steel scrap	234.00	[1]
Graphite electrode	4000.00	[1]
Oxygen	61.00	[1]
Alloying elements	1777.00	[1]
Slag from waste firing	49.29	[10]

Methods S1. Optimization model.

A full model description and mathematical formulation is given for the coupled and soft-linked configurations in the following paragraphs. The variables, sets and parameters are described in tables S1,S2,S3.

Coupled configuration

$$\min_{g,i,\bar{g},\bar{l},\bar{i}} \quad z^{esm} + z^{ind} \tag{1}$$

with:

$$z^{ind} = \sum_{p\in\mathcal{P}}\sum_{n\in\mathcal{N}}\sum_{t\in\mathcal{T}} i_{p,n,t}\left(C_p^{\text{op}} + \sum_{r\in\mathcal{R}} R_{p,r}C_r^{\text{res}}\right) \quad + \sum_{n\in\mathcal{N}}\sum_{p\in\mathcal{P}} \bar{i}_{p,n}C_p^{\text{inv}} \tag{2}$$

$$z^{esm} = \sum_{k\in\mathcal{K}}\sum_{n\in\mathcal{N}}\left(\sum_{t\in\mathcal{T}} g_{k,n,t}\left(C_k^{\text{op}} + \sum_{r\in\mathcal{R}} R_{k,r}C_r^{\text{res}}\right) + \bar{g}_{k,n}C_k^{\text{inv}}\right) + \sum_{e\in\mathcal{E}}\sum_{n,m\in\mathcal{N}} \bar{l}_{e,n,m}C_{e,n,m}^{\text{inv,tr}} \tag{3}$$

Energy balance:

$$D_{e,n,t} = \sum_{p\in\mathcal{P}} i_{p,n,t}\cdot E_{p,e} + \sum_{k\in\mathcal{K}} g_{k,n,t}\cdot E_{k,e} + \sum_{m\in\mathcal{N}}(l_{e,m,n,t} - l_{e,n,m,t}), \quad \forall e\in\mathcal{E}, \forall n\in\mathcal{N}, \forall t\in\mathcal{T} \tag{4}$$

The energy demand includes the exogenous energy demand $D_{e,n,t} = D_{e,n,t}^{\text{exo}}$ consisting of non-industry sectors such aqs transport and buildings and of aggregated industry branches other than steel, cement and HVC production.

Material balance for the energy-intensive materials steel, cement and HVC (The less energy-intensive industry branches are represented as aggregated process heat, electricity and feed-stock demand time series in the energy balances (see equation 4 without the option to switch the process route):

$$D_{m,n,t}^{mat} = \sum_{p\in\mathcal{P}} i_{p,n,t}\cdot M_{p,m} \quad \forall t\in\mathcal{T}, \forall n\in\mathcal{N}, \forall m\in\mathcal{M} \tag{5}$$

Resource constraint: Aggregated limits are set for all other nodes, while additional resource limits are set for the German node (e.g., sustainable biomass availability in Germany)

$$\sum_{p\in\mathcal{P}}\sum_{n\in\mathcal{N}}\sum_{t\in\mathcal{T}} i_{p,n,t}\cdot R_{p,r} + \sum_{k\in\mathcal{K}}\sum_{n\in\mathcal{N}}\sum_{t\in\mathcal{T}} g_{k,n,t}\cdot R_{k,r} \le R_r^{\text{max, tot}} \quad \forall r\in\mathcal{R} \tag{6}$$

$$\sum_{p\in\mathcal{P}}\sum_{t\in\mathcal{T}} i_{p,n,t}\cdot R_{p,r} + \sum_{k\in\mathcal{K}}\sum_{t\in\mathcal{T}} g_{k,n,t}\cdot R_{k,r} \le R_r^{\text{max}} \quad \forall r\in\mathcal{R}, n = \text{'DE'} \tag{7}$$

Emission constraint:

$$\sum_{p\in\mathcal{P}}\sum_{n\in\mathcal{N}}\sum_{t\in\mathcal{T}} i_{p,n,t}\cdot X_p + \sum_{k\in\mathcal{K}}\sum_{n\in\mathcal{N}}\sum_{t\in\mathcal{T}} g_{k,n,t}\cdot X_k \le 0 \tag{8}$$

$$\sum_{p\in\mathcal{P}}\sum_{t\in\mathcal{T}} i_{p,n,t}\cdot X_p + \sum_{k\in\mathcal{K}}\sum_{t\in\mathcal{T}} g_{k,n,t}\cdot X_k \le 0, \quad n = \text{'DE'} \tag{9}$$

The sum of emissions over all nodes must be zero, additionally, the German node must reach zero emissions. Distributed emissions from product decay are assumed to occur in the year of production.

Operational costraints: The operation all technologies is constrained by their capacities. The generation of renewable power plants is further constrained by their time- and location-dependent capacity factors CF, which are defined between 0 and 1 as a fraction of capacity. For all other technologies, $CF_{k,n,t} = 1, \quad \forall n \in \ \forall\, n \in \mathcal{N}, \forall\, t \in \mathcal{T}$.

$$g_{k,n,t} \leq \overline{g}_{k,n} \cdot CF_{k,n,t} \quad \forall\, k \in \mathcal{K},\ \forall\, n \in \mathcal{N}, \forall\, t \in \mathcal{T} \tag{10}$$

$$l_{e,m,n,t} \leq \bar{l}_{e,m,n} \quad \forall\, e \in \mathcal{E}, \forall\, m \in \mathcal{N},\ \forall\, n \in \mathcal{N}, \forall\, t \in \mathcal{T} \tag{11}$$

Capacity constraints: The technology capacities are constrained by location-specific capacity limits.

$$0 \leq \overline{g}_{k,n} \leq \overline{G}^{max}_{k,n} \quad \forall\, k \in \mathcal{K},\ \forall\, n \in \mathcal{N} \tag{12}$$

$$0 \leq \bar{l}_{e,m,n} \leq \overline{L}^{max}_{e,m,n} \quad \forall\, e \in \mathcal{E},\ \forall\, n \in \mathcal{N}, \forall\, m \in \mathcal{M} \tag{13}$$

Transmission between nodes only possible for transmittable carriers (electricity and H_2):

$$\overline{L}^{max}_{e,m,n} = 0, \quad \forall e \in \mathcal{E} \notin \mathcal{E}^{tr}, \forall m, n \in \mathcal{N} \tag{14}$$

We apply the optimal linear power flow method described in the PyPSA documentation[18]. Further constraints are described in the PyPSA documentation (*Storage Unit constraints, Generator constraints, Passive branch flows*). If not said otherwise, the default parameters of PyPSA-Eur are kept, e.g., investment costs, capacity factors, energy carrier limits.

Soft-linked configuration

Industry optimization

$$\min_{i,\bar{i}} \quad z^{ind} + \sum_{p\in\mathcal{P}} \sum_{e\in\mathcal{E}} \sum_{n\in\mathcal{N}} \sum_{t\in\mathcal{T}} i_{p,n,t} \cdot E_{p,e} \cdot \lambda_{e,t} \tag{15}$$

The material balance is the same as in the coupled optimization:

$$D^{mat}_{m,n,t} = \sum_{p\in\mathcal{P}} i_{p,n,t} \cdot M_{p,m} \quad \forall t \in \mathcal{T}, \forall n \in \mathcal{N}, \forall m \in \mathcal{M} \tag{16}$$

Limits on emissions and resources include only the processes associated with industrial production:

$$\sum_{p\in\mathcal{P}} \sum_{t\in\mathcal{T}} i_{p,n,t} \cdot R_{p,r} \leq R^{max}_{r} \quad \forall r \in \mathcal{R}, n = \text{'DE'} \tag{17}$$

$$\sum_{p\in\mathcal{P}} \sum_{t\in\mathcal{T}} i_{p,n,t} \cdot X^{ind}_{p} \leq 0 \quad n = \text{'DE'} \tag{18}$$

Results of industry optimization given to the energy system optimization are industrial energy demand and resource consumption:

$$D^{ind}_{e,n,t} = \sum_{p\in\mathcal{P}} i_{p,n,t} \cdot E_{p,e} \quad \forall t \in \mathcal{T}, \forall n \in \mathcal{N}, \forall e \in \mathcal{E} \tag{19}$$

$$R^{\text{ind}}_{r} = \sum_{p\in\mathcal{P}} \sum_{n\in\mathcal{N}} \sum_{t\in\mathcal{T}} i_{p,n,t} \cdot R_{p,r} \quad \forall r \in \mathcal{R} \tag{20}$$

Energy system optimization

The second step is the energy system optimization, in which the objective includes only energy system technologies:

$$\min_{g,\bar{g},\bar{l}} \quad z^{esm} \tag{21}$$

In the energy balance, equation 21, exogenous final energy demand additionally includes exogenous industrial final energy demand $D^{ind}_{e,n,t}$ derived from the industry optimization,

$$D_{e,n,t} = D^{\text{exo}}_{e,n,t} + D^{ind}_{e,n,t} \tag{22}$$

The emission constraint contains only energy system technologies,

$$\sum_{k\in\mathcal{K}}\sum_{t\in\mathcal{T}} g_{k,n,t} \cdot X_k \leq 0 \quad n = \text{'DE'} \tag{23}$$

and remaining resources not consumed by industry optimization can be used:

$$\sum_{k\in\mathcal{K}}\sum_{t\in\mathcal{T}} g_{k,n,t} \cdot R_{k,r} \leq R^{\text{max}}_r - R^{\text{ind}}_r \quad \forall r \in \mathcal{R}, n = \text{'DE'} \tag{24}$$

.

Variations in soft-linked configuration

Following changes in the soft-linked formulation are made in the variations of the soft-linked optimization:

- Energy carrier price time series: These are an input to the industry optimization in the first step. A comparison of input price and shadow price time series (see fig. S7) shows notable deviations of up to +49 and -27 €/MWh for electricity and -19 €/MWh for synthetic gas occur during winter months, and their input prices are on average 0.31 €/MWh higher (electricity) and 0.91 €/MWh lower (synthetic gas) than the resulting shadow prices. For hydrogen, the deviations appear only from July to November, where input prices are 0.5 to 5 €/MWh lower than output shadow prices at almost every time step. Methanol is constantly 0.79 €/MWh and naphtha 1.45 €/MWh more expensive as input prices to the soft-linked industry model than shadow prices in the energy system model. To test the effect of this inconsistency on the industry optimization results, we (A) set the shadow prices of the energy system optimization as inputs (see fig. S7) and (B) set further energy carrier price variations as inputs (see fig. S9.
- Sectoral resource and emission shares: In this variation, the emission and resource constraints are changed. The share for the industry sector is multiplied with R^{max}_r, and the emission limit is set to the emission balance of the industry sector resulting from coupled optimization, instead of zero.

Secondary material calculation

Secondary material availability for HVC and steel in the year y = 2045 is calculated based on the method of[12] and[13]. Production of materials go to enduses, which result in waste after the lifetime of the respective enduse. The waste in year y is

$waste_y = \sum_{end \in Enduses}(p_{(y-textlifetime_{\text{end}})} \cdot \text{share}_{\text{end}} \cdot \text{recovery}_{\text{end}}$, with p being the production of the material in a specific year. The enduse sectors and their share of produced material, lifetime and recovery rate are given in table S7. We assume that scrap which becomes available in every year is directly consumed, so no waste is remaining from the years before.

We distinguish secondary plastics suitable for mechanical and chemical recycling. For mechanical recycling, packaging waste is suitable[19], since the chemical composition is not changed[4], and a substitution factor of 0.7 for quality loss is applied[14]. For chemical recycling, all plastic waste types are suitable (including sorting residues from mechanical recycling), since the chemical structure is broken down[4]. In the model, the resiudal plastics of mechanical recycling can undergo thermal recycling (recover electricity and heat) or they can be used for chemical recycling, as in the scenarios of[4]. Thus, the residual waste after sorting is available as mixed waste for chemical recycling / energy recovery. As price for packaging waste, we assume a price of 0 €/t as in[17].[4] even take a price of -0.1 €/t[4], thus a reward for handling the waste (They assume costs for landfilling of 0.03 €/kg[4] SI, p.15).

Inputs and assumptions

- Greenfield optimization: The system is optimised for 2045, assuming that existing capacities will be replaced before. An exception is the currently installed capacity for renewables which is set as minimum.

- Overseas H2 imports: We include H2 import via ship transport to LNG terminals at a constant price of 84 €/MWh (taken from[20]). For the countries in scope, overseas pipeline imports are not an option[20].

- Flexibility of technologies: Electricity time series for electric vehicles are an input, but the battery capacities of electric vehicles are not explicitly represented and are assumed to be part of the batteries. Industrial processes are modelled with a flat profile, i.e. are not flexible. Some conversion processes allow flexible operation, e.g., electrolysis (rampable 0% to 100% of capacity), methanolization (50 % to 100 %), Fischer-Tropsch (90 % to 100%), process heat technologies (80 % to 100%), heat pumps and electric heaters and boilers (all 0 % to 100 %), dispatchable electricity generators (all 0 % to 100 %).

- Material demand: We assume the same material production for Germany in 2045 as today (2021). Thus, effects of industry production moving to other countries or changes in material demand are not considered. This is changed in sensitivity analyses. Sources for material production today: steel and cement from the IDEES-2021 database[2]. The database does only give aggregated basic chemicals demand, so we take methanol and HVC production in Germany from[21].

- Spatial scope and resolution: The energy system model comprises one node per country for Germany and countries with a direct power transmission link (12 ”Stromnachbarn” are represented, as in the model version of the Ariadne project[22]). The enhanced industry representation is only applied for the German node, while the simplified industry representation is kept for the other nodes. Process heat provision is endogenous for all nodes (except for the German node in the soft-linked energy system optimization where process heat provision is determined in the previous industry optimization).

- Transmission: Transmission of energy carriers between nodes is only possible for electricity and hydrogen, through the expansion of lines, DC links and pipelines. CO_2, methane, oil /

naphtha, and methanol used at the German node must also be provided by technologies at the German node, and resources cannot be imported from other nodes.

- biomass, biogas, emissions and CCS capacity is limited for Germany. For the other nodes, joint limits are set.
- Temporal resolution and horizon: 3H resolution, 1 year.
- Electricity load: The load scaling factor is set to one, such that today's electricity and space heat load is assumed for standard electricity applications. Additional loads for electric vehicles are added. Electricity demand for other power-to-x technologies is endogenous, e.g., heat pumps, electrolysers, methanolization.
- Process heat options and parameters for different temperature levels are taken from[23] and[24].
- Cement process parameter: The process heat demand and emissions for cement production is calculated based on the clinker content in cement. The clinker content is reduced in cement mixtures with supplementary cementitious materials[25]. The mixtures of different cement sorts are taken from[9] and their process heat demand and emissions are calculated by multiplying their clinker content by process heat demand and emissions for clinker production, which is taken from the IDEES database[2] (data is given for cement, we convert based on the average clinker factor 2020 in Germany, 0.71[10]). Feedstock demand depends on the mixtures in[9] and determines the operational costs of the process.
- HVC process parameter: The parameters for HVC recycling given in[5] are for a 3750 t/a input capacity plant; we convert them to specific costs per kg ethylene output with the ratio of 1:2.39 from[4]. For chemical recycling, we model a pyrolysis process, as in[4]. The pyrolysis unit electrically heats to temperatures of 450 degrees celsius[5] p. 106. Investment costs are summed up for the main steps pyrolysis unit and steamcracker unit[4], and taken from[5] p. 109f. (pyrolysis unit) and from[3] p. 3 (electric steamcracker unit).

References

1. Agora Industrie, FutureCamp, Wuppertal Institut, and Ecologic Institut (2022). Klimaschutzverträge für die Industrietransformation. Aktualisierte Analyse zur Stahlbranche.

2. Joint Research Centre (2024). The JRC Integrated Database of the European Energy System. European Commission. https://data.jrc.ec.europa.eu/collection/id-0110.

3. Gu, J., Kim, H., and Lim, H. (2022). Electrified steam cracking for a carbon neutral ethylene production process: Techno-economic analysis, life cycle assessment, and analytic hierarchy process. Energy Conversion and Management *270*, 116256. https://doi.org/10.1016/j.enconman.2022.116256.

4. Volk, R., Stallkamp, C., Steins, J.J., Yogish, S.P., Müller, R.C., Stapf, D., and Schultmann, F. (2021). Techno-economic assessment and comparison of different plastic recycling pathways: A German case study. Journal of Industrial Ecology *25*, 1318–1337. https://doi.org/10.1111/jiec.13145.

5. Stallkamp, C., Hennig, M., Volk, R., Stapf, D., and Schultmann, F. (2024). Pyrolysis of mixed engineering plastics: Economic challenges for automotive plastic waste. Waste Management (New York, N.Y.) *176*, 105–116. https://doi.org/10.1016/j.wasman.2024.01.035.

6. Stallkamp, C., Steins, J., Ruck, M., Volk, R., and Schultmann, F. (2022). Designing a Recycling Network for the Circular Economy of Plastics with Different Multi-Criteria Optimization Approaches. Sustainability *14*, 10913. https://doi.org/10.3390/su141710913.

7. Schöb, T., Kullmann, F., Linßen, J., and Stolten, D. (2023). The role of hydrogen for a greenhouse gas-neutral Germany by 2045. International Journal of Hydrogen Energy *48*, 39124–39137. https://doi.org/10.1016/j.ijhydene.2023.05.007.

8. Harthan, R.O., Förster, H., Braungardt, S., Bürger, V., Emele, L., Görz, W.K., and et al. (2023). Projektionsbericht 2023 für Deutschland. CLIMATE CHANGE 39/2023.

9. Bosold, D., and Pickhardt, R. (2024). Zemente und ihre Herstellung: Zement-Merkblatt Betontechnik B1 10.2024. Verein Deutscher Zementwerke.

10. Verein Deutscher Zementwerke (2022). Ressourcen der Zukunft für Zement und Beton – Potenziale und Handlungsstrategien.

11. European Cement Research Academy (2022). The ECRA Technology Papers 2022: State of the Art Cement Manufacturing Current technologies and their future development.

12. Kullmann, F., Markewitz, P., Kotzur, L., and Stolten, D. (2022). The value of recycling for low-carbon energy systems - A case study of Germany's energy transition. Energy *256*, 124660. https://doi.org/10.1016/j.energy.2022.124660.

13. Pauliuk, S., Wang, T., and Müller, D.B. (2013). Steel all over the world: Estimating in-use stocks of iron for 200 countries. Resources, Conservation and Recycling *71*, 22–30. https://doi.org/10.1016/j.resconrec.2012.11.008.

14. Meys, R., Frick, F., Westhues, S., Sternberg, A., Klankermayer, J., and Bardow, A. (2020). Towards a circular economy for plastic packaging wastes – the environmental potential of chemical recycling. Resources, Conservation and Recycling *162*, 105010. https://doi.org/10.1016/j.resconrec.2020.105010.

15. Plastics Europe (2020). Circular economy for plastics: Germany - 2020. . https://plasticseurope.org/knowledge-hub.

16. Plastics Europe (2022). The circular economy for plastics: A European overview. . https://plasticseurope.org/knowledge-hub/the-circular-economy-for-plastics-a-european-overview-2/.

17. Meng, F., Wagner, A., Kremer, A.B., Kanazawa, D., Leung, J.J., Goult, P., Guan, M., Herrmann, S., Speelman, E., and Sauter, P. (2023). Planet-compatible pathways for transitioning the chemical industry. Proceedings of the National Academy of Sciences *120*, e2218294120.

18. Brown, T., and et al. (2025). PyPSA: Python for Power System Analysis. . https://pypsa.readthedocs.io/en/latest/.

19. Meys, R., Kätelhön, A., Bachmann, M., Winter, B., Zibunas, C., Suh, S., and Bardow, A. (2021). Achieving net-zero greenhouse gas emission plastics by a circular carbon economy. Science *374*, 71–76.

20. Neumann, F., Hampp, J., and Brown, T. (2024). Energy Imports and Infrastructure in a Carbon-Neutral European Energy System: [preprint]. arXiv:2404.03927.

21. Verband der Chemischen Industrie e. V. (2023). Chemistry 4 Climate - Wie die Transformation der Chemie gelingen kann: Abschlussbericht 2023.

22. Ariadne project (2024). Model documentation: PyPSA. https://ariadneprojekt.de/en/model-documentation-pypsa/.

23. Millinger, M., Hedenus, F., Zeyen, E., Neumann, F., Reichenberg, L., and Berndes, G. (2025). Diversity of biomass usage pathways to achieve emissions targets in the european energy system. Nature Energy *10*, 226–242.

24. Fraunhofer ISI (2024). Direct electrification of industrial process heat: An assessment of technologies, potentials and future prospects for the EU. Agora Industry.

25. Rihner, M.C., Whittle, J.W., Gadelhaq, M.H., Mohamad, S.N., Yuan, R., Rothman, R., Fletcher, D.I., Walkley, B., and Koh, L.S. (2025). Life cycle assessment in energy-intensive industries: Cement, steel, glass, plastic. Renewable and Sustainable Energy Reviews *211*, 115245. https://doi.org/10.1016/j.rser.2024.115245.
